# Local networks of community and healthcare organisations: a mixed methods study

**DOI:** 10.1186/s13104-016-2135-y

**Published:** 2016-07-01

**Authors:** Wendy Kemper-Koebrugge, Jan Koetsenruijter, Anne Rogers, Miranda Laurant, Michel Wensing

**Affiliations:** Faculty of Health and Social Studies, HAN University of Applied Sciences, PO Box 6960, 6503 Nijmegen, The Netherlands; Radboud Institute for Health Sciences, IQ Healthcare Nijmegen, The Netherlands114-IQ Healthcare, Radboud University Medical Center, PO Box 9101, 6500 Nijmegen, The Netherlands; Faculty of Health Sciences, NIHR CLAHRC Wessex, University of Southampton, Highfield Campus Building 67 Room 4017, Southampton, SO17 1BJ UK

**Keywords:** Collaboration, Integrated care, Social network analysis, Mixed methods

## Abstract

**Background:**

Local collaboration of community organisations and healthcare organisations is seen as relevant for the efficiency and efficacy of health and social care because of their potential role in providing social involvement which may reduce the need for the utilisation of formal services. Care organisations connect to each other in different ways, thus comprising an organisational network. This study aimed to describe and explore organisational networks with respect to their activities for people with diabetes mellitus type 2 and potential mechanisms of effective collaboration. Collaboration could include, for example, referring to each other and organising activities together. Potential mechanisms are navigation, negotiation and contagion.

**Methods:**

A mixed methods study was conducted in an urban and a rural area in the Netherlands. The participating organisations were mentioned by a sample of diabetes patients in these regions and by organisations’ representatives in a snowballing procedure. Next a quantitative survey and a semi-structured interview were conducted, including 35 representatives of these local organisations. The social network analysis methods was used to map and characterise the organisational networks based on results from the survey. A thematic analysis of interviews was undertaken to identify how three mechanisms (navigation, negotiation and contagion) were used in the collaboration.

**Results:**

Both interviews and network structures showed evidence of navigation-related mechanisms. Organisations referred patients with diabetes to services within their organisation or to relevant services provided by other organisations. Hardly any negotiation or contagion-related mechanisms were identified. If negotiation between organisations was found, it seemed externally enforced. The density, centrality, and reciprocity in the networks seemed low to facilitate contagion of practices. Some organisations reported actions that could have impacted on contagion. Representatives emphasized the need of network collaboration with local or regional community and healthcare organisations.

**Conclusion:**

The study suggests that navigation to resources is a relevant theme in organisational networks, which could be targeted by interventions. More research is needed to explore the relevance of other network-related mechanisms.

## Background

Diabetes, as other long term conditions, is a major public health problem globally [1, 2]. The challenge is in the provision of resources for the effective management of this long term condition as well as in dealing with pressures on healthcare workforce and healthcare costs [3]. Worldwide policy strategies aim to improve the integration of health and social care systems. Collaboration between community organisations and healthcare organisations is encouraged as a means to improve access, quality, continuity and efficiency of health and social care [4, 5]. Diabetes is an ideal health condition to examine integration of services as the guidelines for a multidisciplinary care team are well described [6]. Collaboration aims to meet the needs of individual patients and to meet the needs of the broader population [7]. Furthermore, strategies to strengthen self-management, social participation, and social support are seen as a means to compensate for insufficient capacity of professional care [8, 9]. Non-professional care providers (e.g. family, friends, neighbours, volunteers) play an important role in day-to-day support of patients and are increasingly involved in the provision of care, but also in the organising of care for their relatives [10, 11]. But the alignment between professional care organisations and informal care (including volunteers) remains a challenge [10, 12, 13], resulting in suboptimal efficiency [14] and inappropriate services [15]. Shifting primary caregiving from a physician centered model to an interprofessional care model is essential to improve care [3, 16]. Nonetheless, current research has not given adequate attention to the consequence of these shifts in care for the organisational context and their influence on voluntary collaboration [7, 17].

Collaboration may emerge in a variety of ways, including enforcement, incentives and leadership. Regardless of these underlying drivers, the resulting networks have potential influence on the emergence and persistence of collaboration. Insight into the networks of organisations can illuminate factors and processes that facilitate the development of effective collaboration [14]. These can be broadly grouped into three categories: *navigation* to relevant resources, including people and organisations; *negotiation* around and coordination of activities, such as providing support, and *contagion* of ideas and practices, such as approaches to care delivery. In the context of regional networks of professional organisations and community-based organisations these network related mechanisms could explain, for instance, referral of individuals to other organisations (navigation), local coordination of activities between different organisations (negotiation), and shared mission and culture (contagion). These three types of network mechanisms will be briefly elaborated below.

Network *navigation* concerns identifying and connecting with relevant existing resources in a network, such as individuals with knowledge or organisations which can provide support. It is an intentional act of an individual to activate social capital [15, 18]. Organisations may develop and use network ties to refer older people to appropriate resources or to enable individuals to navigate themselves. Specific for community and healthcare organisations, cooperation is necessary to make sure every client is transferred to an organisation that matches with the client needs or is supported in an effective way by cooperating organisations. With respect to navigation, organisations make decisions about which organisations to contact and involve in activities. In doing so, they successfully preserve existing relations and develop new relevant ones [14].

*Negotiation* within organisational networks concerns the division of activities across organisations. This implies the shaping and re-shaping of relationships, roles, expectations, means of engagement and communication between network members. The emergence of coordination of activities may be influenced by network characteristics. It is assumed that actions are influenced by organisational interests and strategic interaction. If collaboration has benefits, reciprocity in and continuity of relationships connections are favourable for collaboration [19, 20].

*Contagion* implies that [9, 21] organisations adopt ideas, attitudes and behaviours from other network members on the basis of mechanisms such as imitation and role modelling. These processes are influenced by network characteristics, such as density of the network, the number of closed triads and the level of homogeneity of members in the network. Embeddedness in a network could thus lead to a shared perception and capacity to successfully perform behaviour through shared effort, beliefs, influence, perseverance, and objectives [14, 15]. In these ways, organisations could developed shared goals and approaches in their activities for older people.

These network related mechanisms can be linked to specific, quantifiable network structure characteristics as listed in Table 1 [22–25].

**Table 1:** Assumed links between network related mechanisms and network measures

Network related mechanism	Network measure	Definition	Indication
Navigation	Number of connections		
	1 step reach (min–max)	The mean of the number of organisations in the network that are reached within one step	Higher one step reach indicates more knowledge of possibilities to navigate
	2 step reach (min–max)	The mean of the number of organisations in the whole network that are within reach of two directed steps	Higher two step reach indicates more knowledge of possibilities to navigate
Negotiation	Reciprocity	The extent to which ties are reciprocated	Higher reciprocity indicates more potential coordination of activities
Contagion	Density	The proportion of all possible connections in a network that are actually present	Higher density indicates shared decisions
	Centrality	The degree that a network is organized around a single organisation	Higher centrality indicates key players, which indicates more social influence from key players

In the present study, we analysed whether these three types of network-related mechanisms could be observed in two different regional networks of professional organisations and community based organisations. The study had the following research questions:

- What collaboration exists in networks of community and healthcare organisations in two geographic areas (one rural deprived and one mixed urban)?
- What network-related mechanisms underlying the collaboration between these organisations can be observed?

## Methods

### Study design

We performed a mixed methods descriptive study in two geographical regions, which were cases for the present study. Data was collected from 2013 to April 2014.

### Sampling

Organisations were sampled in two regions. These regions were purposefully selected to reflect an economically deprived rural area and a medium-sized city with both deprived and affluent areas (i.e. a mixed urban area). The rationale for the sampling was based on the aims of a main study, in which this secondary study was embedded [9]. To identify relevant healthcare and community based organisations we followed a stepwise procedure. First, we identified organisations that were reported by 300 patients with diabetes who where included by primary healthcare providers to participate in the main study [9]. Next, from the list with organisations we recruited community organisations offering illness relevant support to patients with diabetes. We included four types of organisations: (a) illness-related organisations (for example primary care practices, home care organisation, physiotherapist practices); (b) health and healthy lifestyle-related organisations (for example exercise related organisations or diet groups); (c) well-being-related organisations (for example community centres, religious organisations); and (d) people’s and patients’ rights organisations (for example a patient council or a diabetes association). Second, in addition to the organisations listed by patients with diabetes, we identified organisations that were relevant for the support to diabetes patients. Third, a snowballing technique was used to identify other relevant organisations. These ‘missing’ organisations were organisations mentioned by two or more interviewees.

### Study population

In each organisation a representative close to the management of day-to-day operations and/or the strategic development of the organisation were included as respondent. Verbal informed consent was obtained. Larger organisations with independent groups in different areas were seen as local branches and treated as separate organisations.

### Data collection

#### Survey

We used a structured self-developed questionnaire for representatives of community and healthcare organisations. This questionnaire was purposefully developed and covered three domains: (a) descriptive information of the organisation and its activities; (b) reach in target group in terms of users of information and participants in activities; and (c) collaboration with other organisations in the local area. Collaboration was asked with the question: ‘Please list any groups/organisations that are important to your own group/organisation’. Also three specifications of collaboration were inventoried: (1) giving and receiving information, (2) organizing activities and finances together, and (3) referring to each other. Answer scales were yes or no. The questionnaire was informed by previous studies and tailored to the focus of this study, in particular to answer research question 1 [26].

#### Semi-structured interview

Next to the questionnaire we conducted a semi-structured telephone interview. The protocol was based on previous studies [26] and tailored to the focus of this study, in particular to answer research question 2. The semi-structured interviews were oriented to exploring network-related processes, from which network related mechanisms could be suggested. Topics were the contribution of the organisation to the needs of patients with diabetes, the influence of the organisation on the health of its patients, involvement of patients in the organisation, influence of economic circumstances on the organisation. All interviews were done by one member of the research team.

The data were collected in the EU-WISE project, an international study on social support and self-management in people with diabetes type 2 [9].

### Ethical approval

Permission was obtained from CMO region Arnhem Nijmegen in The Netherlands, registration number 2013/098.

### Data-analysis

This study used quantitative and qualitative methods to answer the two research questions.

#### Analysis of the questionnaire

Network characteristics were documented and visualized for each of the two regions using specific social network analysis software (Ucinet 6) [24]. The following coefficients were determined (see Table 1): number of network ties, one step reach (the number of organisations in the network that are reached within one step), two step reach (idem but reached within two steps), reciprocity (mutual tie), density (proportion of all possible connections), centralisation (the degree the network is centrated around one organisation).

#### Analysis of the interviews

Semi-structured interviews were analysed using thematic analysis on the concepts of the network-related mechanisms [27]. In the qualitative analysis meaning units (words or sentences) were labelled with codes following the network related mechanisms. The coding was done separately by both researchers (WK and JK). The codes were checked by and discussed by both researchers. Disagreements were solved in a consensus meeting with a third researcher (MW). Codes were reviewed in order to discover patterns in using network-related mechanisms.

Interviewees were quoted as R (rural region) and U (urban region).

For purpose of the analysis we operationalized ‘professional-based organisations’ and ‘volunteer-based’ organisation according to the ratio professionals versus volunteers. This is relevant as community organisations and healthcare organisations in the Netherlands are seldom 100 % professional or 100 % volunteer-based. To get insight into the role of informal caregivers in these organisations, we grouped organisations following the ratio number of paid staff and number of volunteers into three different groups organisations:

*Group 1* two-thirds or more volunteers was considered a volunteer organisation,

*Group 2* two-thirds or more paid staff a professional organisation, and

*Group 3* all other combinations of volunteers and paid staff were considered to be mixed organisations.

## Results

In total 35 representatives from 35 community organisations and healthcare organisations were interviewed. The interviewees were mostly managers, team leaders and coordinating staff. In the urban region the network consisted of more organisations (a total of 22 organisations) compared to the rural region (13 organisations). Compared to the rural region, in the urban region fewer healthcare organisations were identified (one healthcare organisation in the urban region to four healthcare organisations in the rural region) (Table 2).

**Table 2:** Characteristics of participating organisations

	Number of organisations	Consisting of		
		Professional organisations	Mixed organisations	Volunteer organisations
Urban region	22	11 well being		6 well being
		1 healthcare		3 patient rights
				1 lifestyle related
		Total 12		Total 10
Rural region	13	4 well being	2 healthcare	1 well being
		2 healthcare		2 patient rights
				2 life style related
		Total 6	Total 2	Total 5

In the urban region 12 out 22 organisations were ‘professional’ organisations, meaning two-thirds of their staff are paid employees. The majority of the included professional organisations were well-being related organisations (11 out of 12). In the rural region from six out of 13 organisations were professional organisations, and four out of these six were well-being related. Within the volunteer organisations there was greater diversity with regards to professional or volunteer type of organisation.

### Collaboration within networks of organisations

**Fig. 1:**
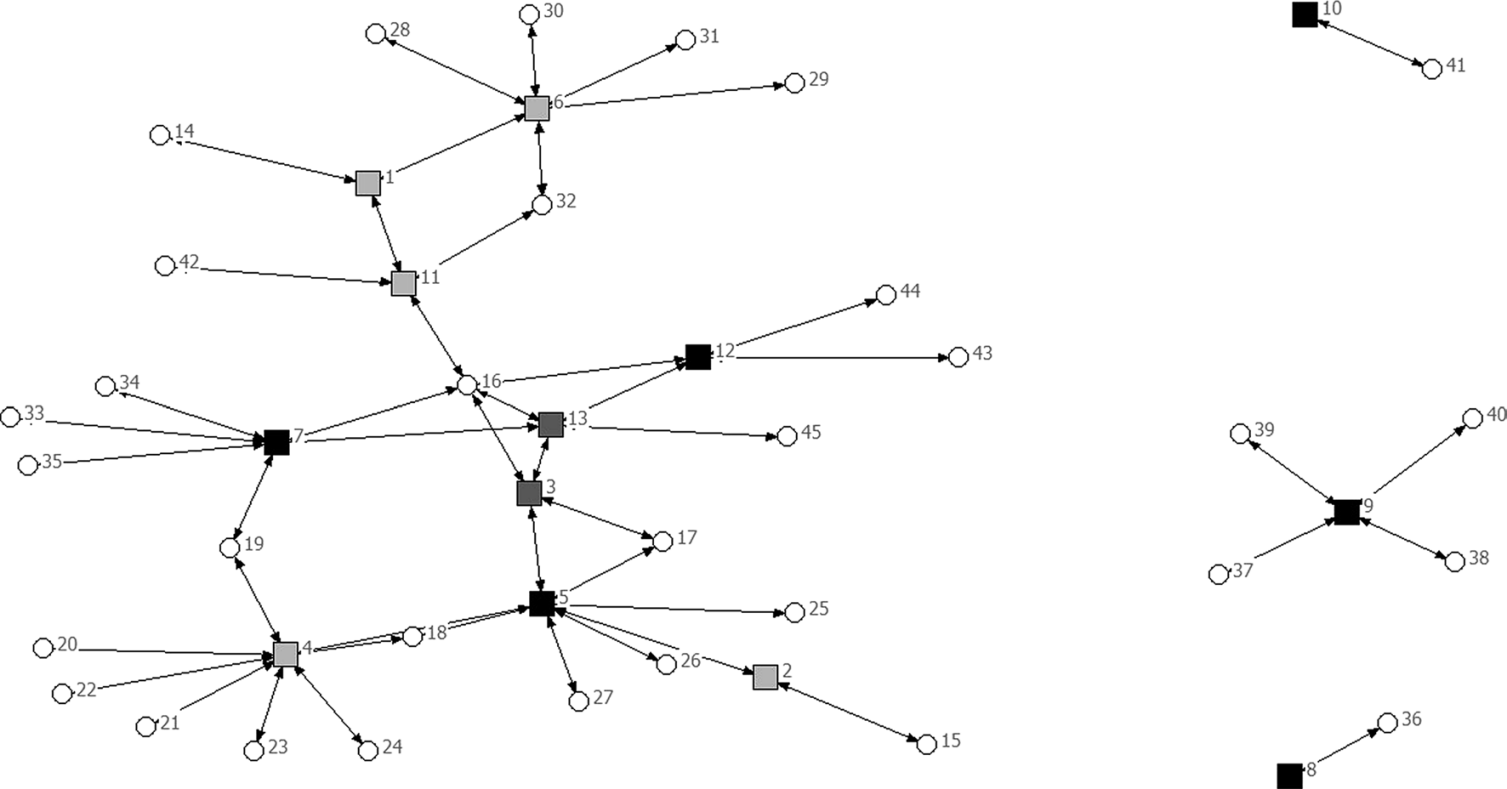
Network of organisations rural region. *Black filled square* professional; *dark grey filled square* mixed; *light grey filled square* volunteer; *Filled circle* mentioned by respondents, not interviewed

**Fig. 2:**
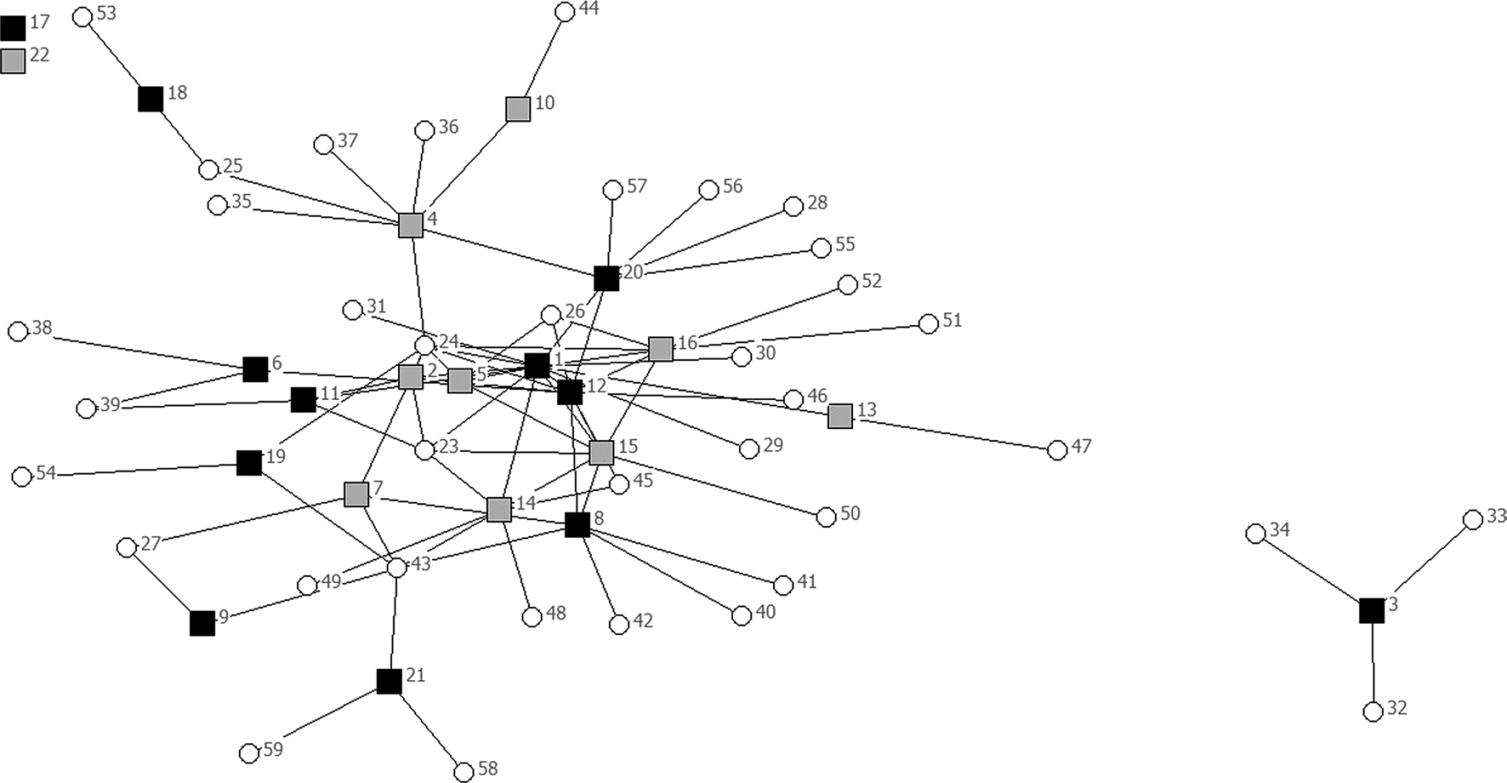
Network of organisations urban region. *Black filled square* professional; *dark grey filled square* mixed; *light grey filled square* volunteer; *Filled circle* mentioned by respondents, not interviewed

The sampled organisations reported links to 69 other organisations in the community and healthcare sector (Figs. 1, 2).

The urban network consisting of 22 organisations contained a larger number of connections compared to the rural network of 13 organisations; 46 connections in the urban network compared to 16 in the rural region (see also Table 3). Density was equal in both regions (0, 1). The urban region had two key players, one professional illness-related organisation and one professional well-being related organisation, with most ties to other organisations in the region. They linked with other professional organisations and other volunteer organisations. This was confirmed by the centrality rate: 36.2 %, compared to 17.4 % in the rural region (see Table 3).

**Table 3:** Network measures

	Urban	Rural
Total number of participating organisations	22	13
Navigation		
Number of connections	46	16
1 step reach (min–max): number of organisations reached in 1 steps	2.09 (0–9)	1.23 (0–3)
2 step reach (min–max): number of organisations reached in 2 steps	6.18 (0–13)	2.46 (0–6)
Negotiation		
Reciprocity	0.17	0.00
Contagion		
Density	0.1	0.1
Centrality (%)	36.2	17.4

In the rural region there were no central actors, but the player in the middle could be seen as a broker occupying a structural hole [28]. The broker, a home care (professional) organisation, seemed to occupy the position between two clusters: a cluster consisting of volunteer organisations and a cluster of professional or mixed organisations. The separate clusters, not linked to the main network of organisations, consisted in the urban region of a well-being related organisation and in the rural region a general practice (the only one in the sample).

Periodic multidisciplinary meetings, the simplest form of collaboration (in terms of the restricted function of giving and receiving information) was most common, but only presented in half of all organisations (16 organisations out of 35 organisations in total). Periodic multidisciplinary meetings facilitated projects in order to create a broad range of services. This was, however, only identified by three organisations. When organisations engaged in organising activities together, they also tended to refer patients to each other. Referring did not automatically imply many network ties; many organisations who refer used limited network partners. Collaboration did not relate to finances, hampering joint action with network members. In the case collaboration in finances was present, it took the form of mutual exchange of professionals without sending invoices.

Due to the economic crisis, organisations in both regions indicated that more cooperation is necessary in the near future. Services should merge or lapse. “*The health system provides enough care, but could be more efficient. There are too many agents in the system and this causes delays in offering support*” (R4).

The main task for the near future is to integrate the network: “*professional and informal care should be more aligned. We have to train professionals to involve the network. Due to less finances, we can rely less on professional care. Professional caregivers are not used to this*” (U31).

### Presence of network-related mechanisms in collaborating with other organisations

Table 3 shows output of the network measures, related to network related mechanisms.

Opportunities for *navigation* seemed to be present in both regions. The number of connections and the number of organisations that could be reached in one step or two steps seemed larger in the urban region than in the rural region. This was confirmed in the interviews, which showed that 12 out of 22 organisations explicit using navigation in the urban region, compared to four out of 13 in the rural region.

Navigation took the form of referring patients to support in their own organisation and to services offered by organisations within one step reach. Organisations indicated to match volunteers and people who need support in order to counter loneliness or stimulate them to attend activities they organise.

The use of network seemed mostly directed to reaching potential patients: “*we reach groups through the network in the neighbourhood and through personal contact. Also we reach people through our network as organisation*”(U20).

Almost all organisations referred to difficulties in reaching hard to reach groups. “*immigrants think their children should help, not strangers. We are locals; interaction with people from abroad is more difficult. Secondly there is the language barrier*” (U23). “*We use immigrants to reach immigrants*”(R38).

In reaching patients, organisations reached out to other organisations, but mostly to organisations within one or two steps of reach. For example, one coordinator only mentioned the other parts of his organisation as a network, although he encountered difficulties in reaching his target population.

Professional organisations tended to offer the support themselves instead of navigating to possibilities in informal care: “*because we have fewer paid carers, we can offer less help*” *(U17)*. On the other hand, professional organisations considered themselves as coordinating informal care because of their knowledge and the attributed absence of the capacity to self-managein patients “*the professional coordinates and has a role in developing the network. The transition is from providing to ensuring support.*” (R7, R38). Awareness of the transition extends to patients: “*people should be aware that they have to organize things for themselves*” (U44).

Support is given mostly to people who are able to participate or navigate: “*people who navigate themselves get support*” (U24). “*Not all groups are involved, we reach the groups who participate*”. People who are alone, stay at home, have difficulties making contact or don’t ask for help, are difficult to reach. Organisations did not purposefully make the network accessible for these hard to reach individual patients suggesting the reinforcement of inequalities of access given that support seemed to be directed at those who were already richer in network contact with others.

Navigation was often directed at reaching patients, but organisations expressed difficulty with developing network ties with other organisations: “*there are many supporting organisations, but finding the right ones is difficult*” *(R8).*

We found a few signs of *negotiation* mechanisms. The reciprocity rate in the urban region was low with only 17 % of network ties identified as being reciprocal. In the rural region, reciprocity was absent. In the interviews negotiation was visible with four organisations in the urban region compared to one organisation in the rural region. In the rural region this negotiation involved the professional who coordinates the social network around a client (R7).

Task reallocation took place in the urban region within an organisation, making a shift from professional to informal care through buddy projects: “*people with a disorder are trained so they can support other people from experience*” (U6). Task reallocation between organisations was external driven: “*the local government asks us to take over home care. This causes conflicts*” (U25) and “*we should involve the informal carer more in the network*” (U31).

*Mechanisms for contagion* of ideas and practice were rarely evident. Out of the possible network ties, 10 % was actually present, so possibilities for influencing were low. Also taking into consideration that only 17 % of ties were reciprocal, ties seemed weak for transmission. Social influence from key players to other organisations was more likely in the urban region, but not confirmed in the interviews.

Organisations did not report to use contagion deliberately. When they quoted action, which could be considered strategic for contagion, this action was directed at patients and not intended as contagion. However, activities in which contagion between patients can occur, was cited. Organisations detected communities that support their patients, stimulated interaction between patients and brought people together in peer groups and activities: “*we involve family and friends and stimulate patients to look for sponsors and support in self*-*help groups*” (U27). A religious organisation indicated that their church community is the base on which they organize activities: “*people can meet and support each other in social activities we organise in this community*” (U19). Sport organisations suggested that sport or movement is a good way for people to get into contact with other people: “*sport is a means to ameliorate health and enlarge social contacts,sport enhances self confidence*” (R3, R11).

## Discussion

The value of this study is in providing insight into local networks of community and healthcare organisations that facilitate access to resources and sources of support for people with diabetes mellitus type 2. On account of the organisations representatives and in the observed network structures, we mainly found evidence of navigation-related mechanisms. These were relevant to reach the target groups and to refer individual patients to appropriate services. We found few clues for negotiation or contagion-related mechanisms in these organisational networks which are more likely to result in the provision of or mobilisation of tangible resources of support. Volunteer or mixed organisations did not differ in their way of using the network from professional organisations. A study of voluntary groups in Manchester confirms and nuances these results [29]; network utilisation by volunteer groups reflected assumptions of the group’s function for its members. Network ties were developed advertising or facilitating the own voluntary group. Externally funded groups developed more collaboration with other organisations supporting their aim to provide a range of services. In the urban region in our study the two central players also perform this role in the system.

Connections between health services and community and healthcare organisations were sparse in our results. The Manchester study detailed frustration at opportunities that had not developed with health services, because of differences in approaches to health [29]. This may limit the role that voluntary organisations can play in providing a more public health orientated care system.

A key theme in a scoping review linking health services to community and healthcare organisations was that individual health professionals played an important role in referring patients by providing credibility or legitimacy of the service for the patients [30]. Connections between health services and community and healthcare organisations could thus add to effective use of the total network.

Network related mechanisms were not explicitly asked about, but derived from answers to questions concerning each organisations contribution to clients. The participants did not relate these questions to terms of active network management. Studies on local strategic partnerships in England [31] state that partnerships often seem to be designed to avoid any loss of power by their members rather than pooling power and resources. Organisations function on their own histories, cultures and preoccupations and are not necessarily managed on collaboration. Successful partnerships were outcome-focused, aligned relationships between different levels and responded to service users’ needs and concerns.

Navigation to relevant organisations offers the clearest indication to the design requirements of network-related interventions, such as online platforms that present local support organisations. The absence of suggestions for negotiation and contagion mechanisms in organisational networks may suggest that further research is needed to explore their potential for enhancing these, before interventions to improve collaboration in networks and to integrate professional and informal care, can be designed [32]. For example, a two-step reach could be handled by an organisation in choosing which network ties to deepen in direct contact and which network ties to negotiate with other organisations.

The study has important implications for practice and policy. In recent years, many activities regarding social support for health and well-being have been decentralized towards the municipal level in the Netherlands [33]. This enlarges the role and possibilities for organisations to use the network, especially since decentralized systems may prove difficult for individual patients to gain insight into their entitlements. Community and healthcare organisations should shift from the narrow use of the network serving their own interests and modus operandi [19] to using the network to supplement or as an alternative to what can be provided through professional care [8, 9]. There would potentially be more utility for individual patients to access the total network. As a conduit for mobilising support resources this would reinforce individual capacities for self- management. For professional or mixed organisations this shift is requires even more, given that professionals in this study identified their role as crucial in delivering support or coordinating the network. Research on how to enlarge the impact of informal care in these network constellations could add to understanding of this integration of networks and the potential contribution to the access, quality, continuity and efficiency of health and social care.

A strong aspect is the mixed methods approach of this study as quantitative methods were corroborated by qualitative methods. Nonetheless, also some limitations should be mentioned. First it was hard to define the boundaries of the network, which is a common problem in network mapping [34]. To solve this issue, the sampling provided an extra check for relevant organisations, but some organisations could still have been missed if they were not mentioned by patients or representatives [34, 35]). Second, network sizes seemed to be small, but other studies suggest that healthcare networks often have similar sizes [36]. Also the measured density is common comparable to other studies. Third the participants in the study were managers and coordinating staff, who can be expected to oversee the connections with other local organisations. But, the low degree of reciprocity in the identified connections may raise concern on the validity of the reported connections. This could have been triggered by the phrasing of the question in terms of important organisations or by the fact that respondents had to remember organisations themselves. Last the generalizability of findings was enhanced by using a pre-defined framework, but is uncertain given the small number of cases.

## Conclusions

The study suggests that navigation to resources is a relevant theme in organisational networks for linking people into extended sources of social support, which could be targeted by interventions. More research is needed to explore the relevance of other network-related mechanisms.
